# Impact of a Resident-Built, ADA-Aligned Epic Template on Guideline-Based Diabetes Care Processes and HbA1c in a Teaching Clinic: A Quality Improvement Initiative

**DOI:** 10.51894/001c.163957

**Published:** 2026-08-01

**Authors:** Tanisha Vora, Marena Dib, Paige Misner, Jacky Duong

**Affiliations:** 1 Trinity Health Oakland Hospital

## 03

### BACKGROUND

Comprehensive diabetes preventive care remains inconsistently delivered and documented in U.S. practice. Recent studies show that 8.2% of adults with diabetes received none of the recommended preventive services in 2020, annual professional foot examination was reported in only 71.6%, and albuminuria screening detects only about one-third of prevalent cases. These may be greater in resident-run clinics because of rotating providers and workflow variability. To improve physician and resident adherence, we implemented a resident-developed, ADA guideline–based Epic template to standardize documentation and completion of key preventive care elements.

### OBJECTIVES

To evaluate whether implementation of a resident-developed ADA-aligned Epic template improves physician and resident adherence to guideline-based diabetes care processes and its short-term impact on HbA1c and diabetes-related acute care utilization.

### METHODS

We performed a single-center quality improvement pre–post evaluation at Henderson Clinic (Trinity Health Oakland), a resident-run ambulatory clinic. Adults (≥18 years) with type 2 diabetes and paired hemoglobin A1c (HbA1c) measurements in the pre-intervention period (July 1–December 31, 2023) and post-intervention period (January 1–December 31, 2025) were included. The intervention consisted of implementation of the ADA-aligned Epic template with resident education. Process measures included documentation or completion of eye examination, foot examination, estimated glomerular filtration rate (eGFR), and urine microalbumin. Provider adherence was assessed by template utilization. Clinical outcomes included within-patient HbA1c change and diabetes-related ED visits or hospitalizations. Paired t-tests and Fisher’s exact tests were used.

### RESULTS

Ninety-one patients had paired HbA1c measurements. Template utilization was 100% (91/91), indicating complete resident adoption. Mean HbA1c decreased from 7.33% to 7.21% (mean change −0.12; 95% CI, −0.55 to 0.31; *p*=0.585). Guideline-based care processes improved significantly: eye examination documentation 4/91 (4.4%) vs 19/91 (20.9%) (*p*=0.00132), foot examination 30/91 (33.0%) vs 87/91 (95.6%) (*p*=1.97×10⁻¹²), eGFR documentation 17/91 (18.7%) vs 77/91 (84.6%) (*p*=9.24×10⁻¹³), and urine microalbumin 20/91 (22.0%) vs 55/91 (60.4%) (*p*=2.08×10⁻⁷). Diabetes-related ED visits or hospitalizations did not differ significantly.

### CONCLUSIONS

A resident-developed ADA-guideline–based Epic template achieved complete adoption and significantly improved documentation and completion of key diabetes preventive care processes. Although short-term HbA1c and acute-care utilization were unchanged, the intervention improved reliability of comprehensive diabetes care delivery in a resident-run clinic.

